# Enhancing enteric pathogen detection: implementation and impact of multiplex PCR for improved diagnosis and surveillance

**DOI:** 10.1186/s12879-024-09047-z

**Published:** 2024-02-07

**Authors:** Jad Mohtar, Hiba Mallah, Jean Marc Mardirossian, Rana El-Bikai, Tamima El Jisr, Shatha Soussi, Rania Naoufal, Gabriella Alam, Mira El Chaar

**Affiliations:** 1 Faculty of Health Sciences, University of Balamand, Beirut, Lebanon; 2 Clinical Laboratory Department, Makassed General Hospital, Beirut, Lebanon; 3 Clinical Laboratory Department, Saint Georges Hospital University Medical Center, Beirut, Lebanon; 4 Mayo Clinic Discovery and Translational Polycystic Kidney Disease Center, Florida, USA

**Keywords:** Enteric pathogens, Prevalence, Gastroenteritis, Multiplex PCR, Hospitalization rate

## Abstract

**Background:**

Syndromic surveillance of acute gastroenteritis plays a significant role in the diagnosis and management of gastrointestinal infections that are responsible for a substantial number of deaths globally, especially in developing countries. In Lebanon, there is a lack of national surveillance for acute gastroenteritis, and limited data exists regarding the prevalence of pathogens causing diarrhea. The one-year study aims to investigate the epidemiology of common gastrointestinal pathogens and compare our findings with causative agents of diarrhea reported by our study collaborative centers.

**Methods:**

A multicenter, cross-sectional study was conducted over a one-year period. A total of 271 samples were obtained from outpatients and inpatients presenting with symptoms of acute gastroenteritis at various healthcare facilities. The samples were then analyzed using Allplex gastrointestinal assay that identifies a panel of enteric pathogens.

**Results:**

Overall, enteropathogens were detected in 71% of the enrolled cases, 46% of those were identified in patients as single and 54% as mixed infections. Bacteria were observed in 48%, parasites in 12% and viruses in 11%. Bacterial infections were the most prevalent in all age groups. Enteroaggregative *E. coli* (26.5%), Enterotoxigenic *E. coli* (23.2%) and Enteropathogenic *E. coli* (20.3%) were the most frequently identified followed by *Blastocystis hominis (15.5%) and* Rotavirus (7.7%). Highest hospitalization rate occurred with rotavirus (63%), Enterotoxigenic *E. coli* (50%), *Blastocystis hominis* (45%) and Enteropathogenic *E. coli* (43%). Enteric pathogens were prevalent during summer, fall and winter seasons.

**Conclusions:**

The adoption of multiplex real-time PCR assays in the diagnosis of gastrointestinal infections has identified gaps and improved the rates of detection for multiple pathogens. Our findings highlight the importance of conducting comprehensive surveillance to monitor enteric infections. The implementation of a syndromic testing panel can therefore provide healthcare professionals with timely and accurate information for more effective treatment and public health interventions.

**Supplementary Information:**

The online version contains supplementary material available at 10.1186/s12879-024-09047-z.

## Introduction

Enteric infections cause significant morbidity and mortality worldwide with an estimate of more than one million people dying each year of infectious gastroenteritis [1, 2]. The emergence of gastrointestinal infections differs markedly between developed and developing countries, shaped by factors such as sanitation, healthcare infrastructure, food safety standards, economic disparities, education, and climate [3, 4]. It is estimated that viruses are the leading cause of acute gastroenteritis followed by bacteria and parasites [3]. Most cases of gastroenteritis may resolve without treatment within days, although persistent or severe symptoms may lead to hospitalization; young children, elderly and immunocompromised patients are at higher risk of complications [5–8].

Diarrhea can be categorized based on duration and clinical features. Acute diarrhea, lasting less than two weeks, is often caused by infections. Persistent diarrhea, lasting 2–4 weeks, may be associated with chronic conditions. Chronic diarrhea, lasting over four weeks, is linked to ongoing medical problems [9]. Inflammatory diarrhea involves blood and inflammation and is indicative of conditions like inflammatory bowel disease, while non-inflammatory diarrhea is watery and associated with viral infections [10]. Dysentery is characterized by severe diarrhea with blood and mucus, commonly caused by invasive infections. Categorizing of diarrhea is essential for the diagnosis and treatment of gastroenteritis.

Recent advancements in molecular diagnostic technology with multiplexing capabilities have made possible syndromic nucleic acid amplification for the diagnosis of many causative agents, which may not be detected by conventional methods [11–21]. The application of these assays has significantly reduced the turnaround time of detection hence improving the value of an etiologic diagnosis with respect to the patients’ management [15, 17, 22, 23].

In Lebanon, there is a lack of national surveillance for acute gastroenteritis and limited data about the prevalence of pathogens causing diarrhea. Most stool investigation protocols in Lebanon have a limited scope of screened pathogens in acute gastrointestinal infections; most diagnostic laboratories still rely on conventional methods for the identification of pathogens such as enzyme immunoassays for the detection of Rotavirus, Adenovirus, and *Clostridium difficile* toxin, bacterial culture for the detection of *Salmonella* and *Shigella*, and direct wet mount microscopy for parasites [24, 25]. The one-year study aims to investigate the epidemiology of common gastrointestinal pathogens and compare our findings with causative agents of diarrhea as reported by our study collaborative centers.

## Methods

### Sample and data collection

The study was a cross-sectional study that was conducted in Lebanon between June 2020 and May 2021. Patients were recruited using a convenience sample of 271. The samples were collected consecutively from two major tertiary care centers and one outpatient clinic in Beirut, Lebanon. Specifically, we obtained 141 samples from Makassed General Hospital, 80 samples from Saint George hospital and 50 samples from Doctor Center outpatient center.

The inclusion criteria were the following: patients who had diarrhea with the passage of three or more loose or liquid stools per day in the 24 h; patients with gastroenteritis accompanied with fever or experiencing bloody diarrhea. Exclusion criteria for selection included patients with an ongoing bowel disease (bowel cancer, inflammatory bowel disease, ulcerative colitis, irritable bowel syndrome, or Crohn’s disease); patients experiencing gastroenteritis symptoms following medication, alcohol consumption, or pregnancy; patients with any known chronic illness with symptoms of diarrhea other than ones listed above. Fresh samples were collected, and each sample was divided for processing. A portion underwent conventional processing at the tertiary care centers, while the remaining part was stored at -80 °C for subsequent analysis.

### Data collection

The participation of the patients was voluntary, verbal consent was obtained from the patients or their guardians for their enrolment in this study. Clinical specimens’ and data collection methods were approved by the institutional review board of Makassed General Hospital. A standardized questionnaire was filled by the laboratory staff which included demographic data, specimen description, date of collection, presence of WBC, RBC, and the laboratory microbiology results. For the inpatients’ cases, additional information was collected including admission status, cause of hospitalization, date of admission and discharge, clinical symptoms, and list of antibiotics used during hospitalization. The questionnaires were completed except for 21 (7.7%) that were missing. Age and gender were missed in 23.6% and 6.6%, respectively. WBC counts were reported in 40% and RBC in 11%.

### Nucleic acid extraction

QIAamp DNA Mini Kit (Qiagen, Hilden, Germany) was used for the extraction of total nucleic acid from stool according to the manufacturer’s protocol. A FLOQ swab was used to collect stool from the collected specimen and suspended in 1 ml of stool lysis buffer (ASL buffer) and incubated for 10 min at room temperature. A total of 200 µl of each sample was then extracted. Internal controls provided by the manufacturer were added to all samples prior to extraction. Nucleic acid were concentrated in 50 µl of elution buffer. A total of 5 µl of nucleic acid was used for each reaction.

### Detection of diarrheal pathogens by real time PCR

Allplex™ full gastrointestinal assay is a multiplex one-step real-time RT-PCR assay that detects and identifies 25 gastrointestinal pathogens (Table S1) including 13 bacteria, 6 viruses, and 6 parasites simultaneously. Based on Seegene’s proprietary Multiple Detection Temperature (MuDT™) technology, this assay reports multiple Ct values of each pathogen in a single channel. Multiplex PCR was performed following the manufacturer’s recommendations on a CFX96 Real-Time Detection System (Bio-Rad, Hercules, CA, USA). The multiplex real-time PCR has high sensitivity and specificity by utilization of Dual priming oligonucleotide (DPO)-based real-time RT-PCR (DPO™) and Tagging Oligonucleotide Cleavage Extension (TOCE™) technologies. The PCR reaction was performed under the following cycling conditions: 20 min at 50 °C for 1 cycle; 15 min at 95 °C for 1 cycle; 10 s at 95 °C, 1 min at 60 °C and 30 s at 72 °C for 45 cycles; 10 s at 95 °C, 44 more times. Seegene Viewer Software (Seegene Inc. Seoul, Korea) was used for detection and data analysis. Samples were reported as positive at a cycle threshold value of < 40.

SARS-CoV-2 was detected by a fluorescent PCR Kit (Maccura Biotechnology, USA), which is a multiplex PCR assay that detects three genes ORF1ab, N and E genes. The kit was used according to the manufacturer’s instructions.

### Statistical analysis

Data analysis was performed using SPSS (version 28.0). Descriptive analysis were used to calculate the mean, median, frequencies and percentages of the population under study. Prevalence was calculated to indicate the number of subjects carrying the enteric pathogen. Categorical variables such as seasonality were evaluated by Chi-square test. All *P*-values less than 0.05 were considered statistically significant. Missing values were excluded from the analysis.

## Results

### Demographic characteristics of the sample

A total of 271 patients aged between 0 and 99 years were enrolled in this study. Their mean and median age was 38 years (SD = 32.5) and 49 years (SD = 32.5), respectively. A total of 47.6% were males and more than 50% of the cases were outpatients (56.1%). The age was categorized into 18 groups [20]; The two largest groups involved in this study were the participants with age group less than 5 years (*N* = 57/207, 27.5%) and more than 60 years (*N* = 72/207, 34.8%) (Figure S1).

### Prevalence of enteropathogens

Overall, enteropathogens were detected in 71% (*N* = 192) of the 271 patients enrolled cases. Bacteria were observed in 48% (*N* = 130), parasites in 12% (*N* = 32) and viruses in 11% (*N* = 30). Bacterial infections were the most prevalent in all age groups. A total of 79 samples were negative (29%).

**Fig. 1:**
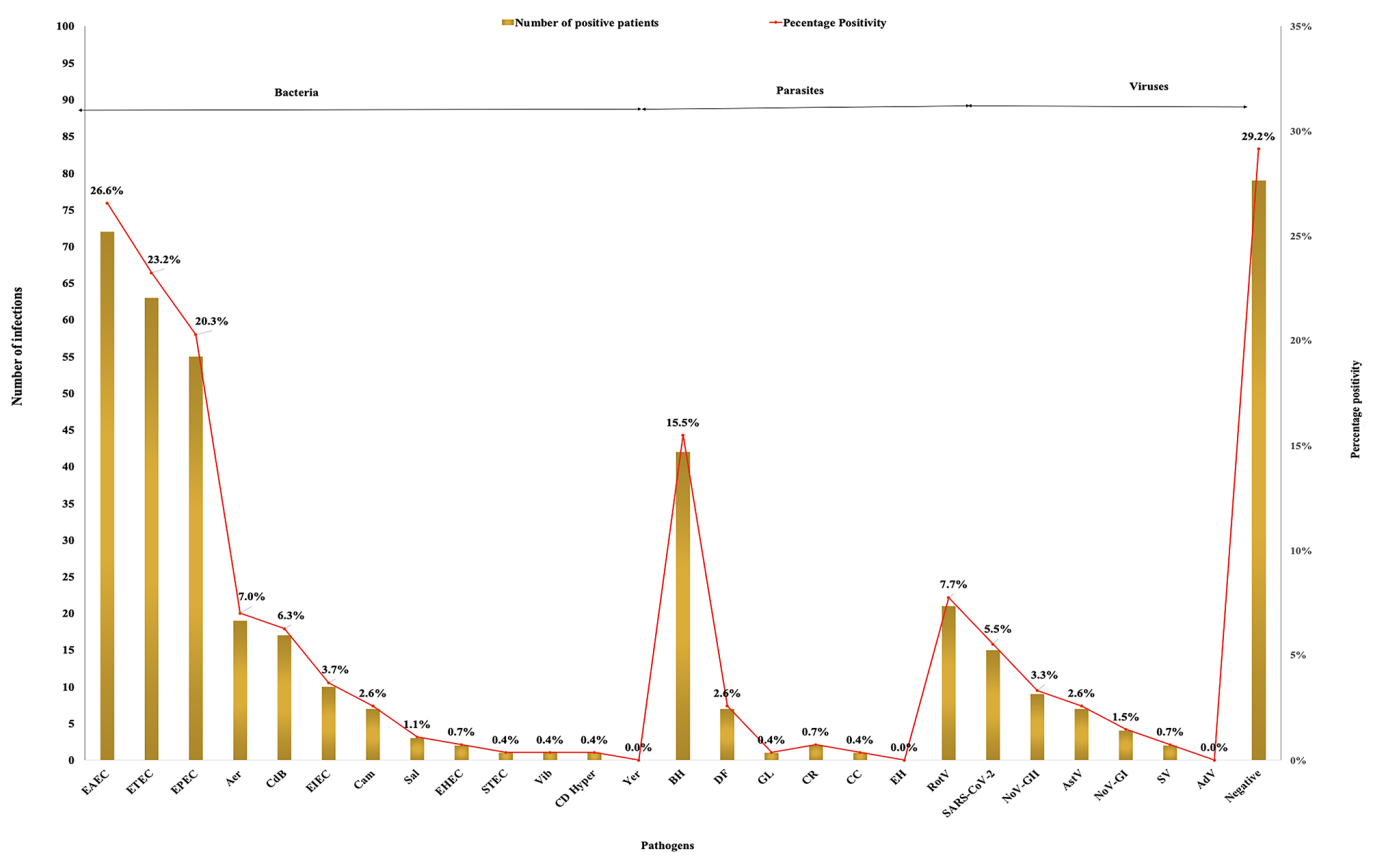
Prevalence of enteropathogens detected in patients with acute diarrhea during a 1-year period

**Fig. 2:**
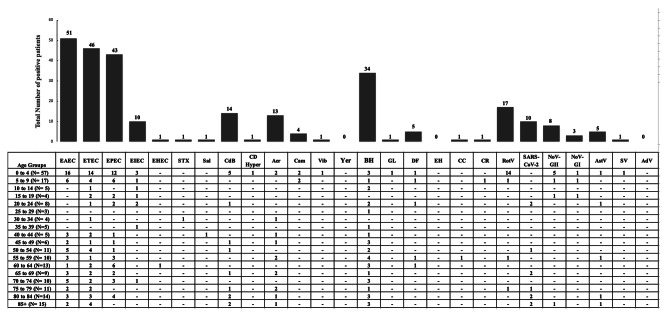
Distribution of infection rates in relation to the age of patients. The number of patient infected are represented in the y-axis, and the x-axis is described by tested pathogen. Positive patients for an infection with no reported age were not included in the analysis

Enteroaggregative *Escherichia coli* (EAEC) (26.5%, *N* = 72), Enterotoxigenic *Escherichia coli* (ETEC) (23.2%, *N* = 63) and Enteropathogenic *Escherichia coli* (EPEC) (20.3%, *N* = 55) were the most frequently identified pathogens followed *by Aeromonas spp* (Aer)(7%, *N* = 19), *Clostridium difficile* (CdB)(6.3%, *N* = 17), Enteroinvasive *Escherichia coli* (EIEC)(3.7%, *N* = 10) and *Campylobacter spp* (Cam)(2.6%, *N* = 7) (Fig. 1). All other tested bacterial pathogens such as *Salmonella* (Sal), Shiga toxin-producing *Escherichia coli* (STEC). *Vibrio cholerae* (Vib), Hypervirulent *Clostridium difficile* (CD Hyper), and *Yersinia entercolitica* (yer) were less commonly identified. Among the parasitic infections tested, *Blastocystis hominis* (BH) (15.5%, *N* = 42) and *Dientamoeba fragilis* (DF) (2.6%, *N* = 7) were the most prevalent (Fig. 2). As for viruses, rotavirus (RotV) (7.7%, *N* = 21) and SARS-CoV-2 (5.2%, *N* = 14) were the two most common causes of viral gastroenteritis.

Among the reported findings from the health centers involved in our study, utilizing conventional diagnostic methods, *Salmonella* culture identified three positive cases, constituting 1% of the examined samples. *Shigella* culture yielded zero positive cases, indicating the absence of *Shigella* among the provided data. The detection of Rotavirus, assessed through a rapid antigen assay, revealed 15 positive cases, constituting 6% of the samples. Adenovirus culture, on the other hand, yielded zero positive cases. Additionally, direct wet mount examination for parasites identified 32 positive cases, representing 12% of the samples. Similarity was observed for *Salmonella, Shigella*, Rotavirus, and Adenovirus between conventional and multiplex PCR results. However, several parasites, particularly Blastocystis hominis, and various *E. coli* subtypes, as well as Norovirus and SARS-CoV-2, were missed by conventional methods.

Pathogens were also categorized according to age since the incidence of pathogens can vary accordingly. Children aged less than 4 years old (*N* = 57) were predominantly infected with RotV (24.5%, *N* = 14, P value < 0.00001), EAEC (28%, *N* = 16, *P* value = 0.479), EPEC (21%, *N* = 12, *P* value = 0.951) and ETEC (24.5%, *N* = 14, *P* value = 0.617) (Fig. 2). *Blastocystis hominis* infections showed no age specific predominance and it occurred in all age group.

### Prevalence of single infection versus hospitalization rate

**Fig. 3:**
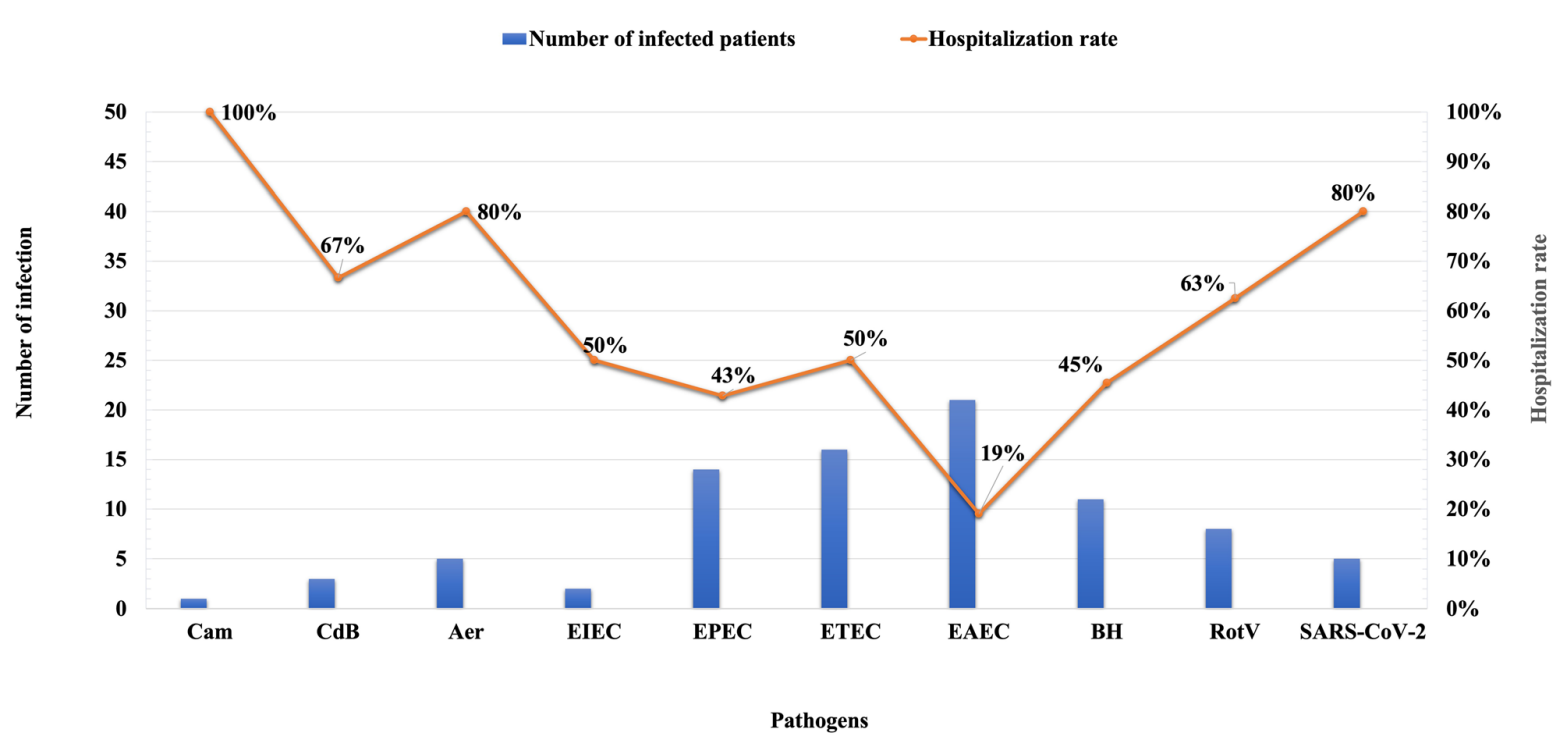
Hospitalization rate of enteric pathogens

Monoinfection occurred in 88 patients (32.5%) with the highest hospitalization rate occurring with RotV (63%; 5 patients were hospitalized out of 8), ETEC in 50% (8 out of 16), BH in 45% (5 out of 11), and EPEC in 43% (6 out 14) (Fig. 3). Hospitalization rates were observed with patients infected with different type of pathogens, however the infection rate for these pathogens was low (Fig. 3).

### Prevalence of coinfections

**Fig. 4:**
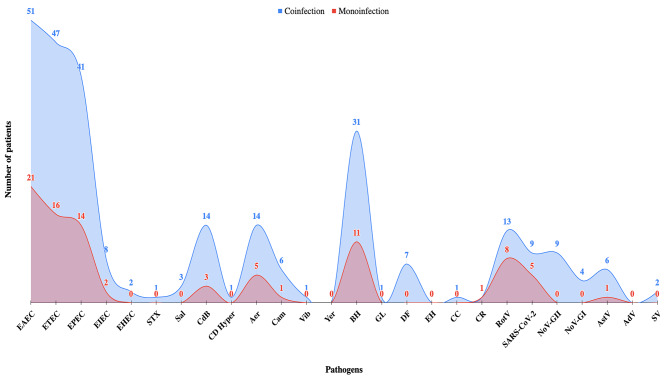
Rate of monoinfections and coinfections for each pathogen

There were 76 different types of combined coinfections involving various microorganisms with the following combinations: Bacterial-bacterial (33%, *N* = 25), Viral-bacterial (29%, *N* = 22) and parasitic-bacterial (22%, *N* = 17). Among the total 104 patients, coinfections were seen as two pathogens (57%, *N* = 59), three pathogens (31%, *N* = 32), four pathogens (7%, *N* = 7), five Pathogens (5%, *N* = 5) and six pathogens (1%, *N* = 1) (Table S2). Coinfection was prominent in EAEC, ETEC, EPEC, CdB, Aer, BH, DF, RotV, NoV-GII and AstV infected cases (Fig. 4). ETEC-EAEC and EPEC-EAEC accounted for the highest percentage of all coinfections, 14% (*N* = 11/76) and 4% (*N* = 4/76), respectively (Table S2).

There was no correlation between coinfection with the high number of pathogens and hospitalization rate (*P* value = 0.603). High WBC in stool was only seen in 50% of the patients who have a coinfection of three pathogens and above. No correlation was seen between specific symptoms, WBC count in stool and specific pathogen (*P* values > 0.05).

### Seasonality of the enteropathogens

**Fig. 5:**
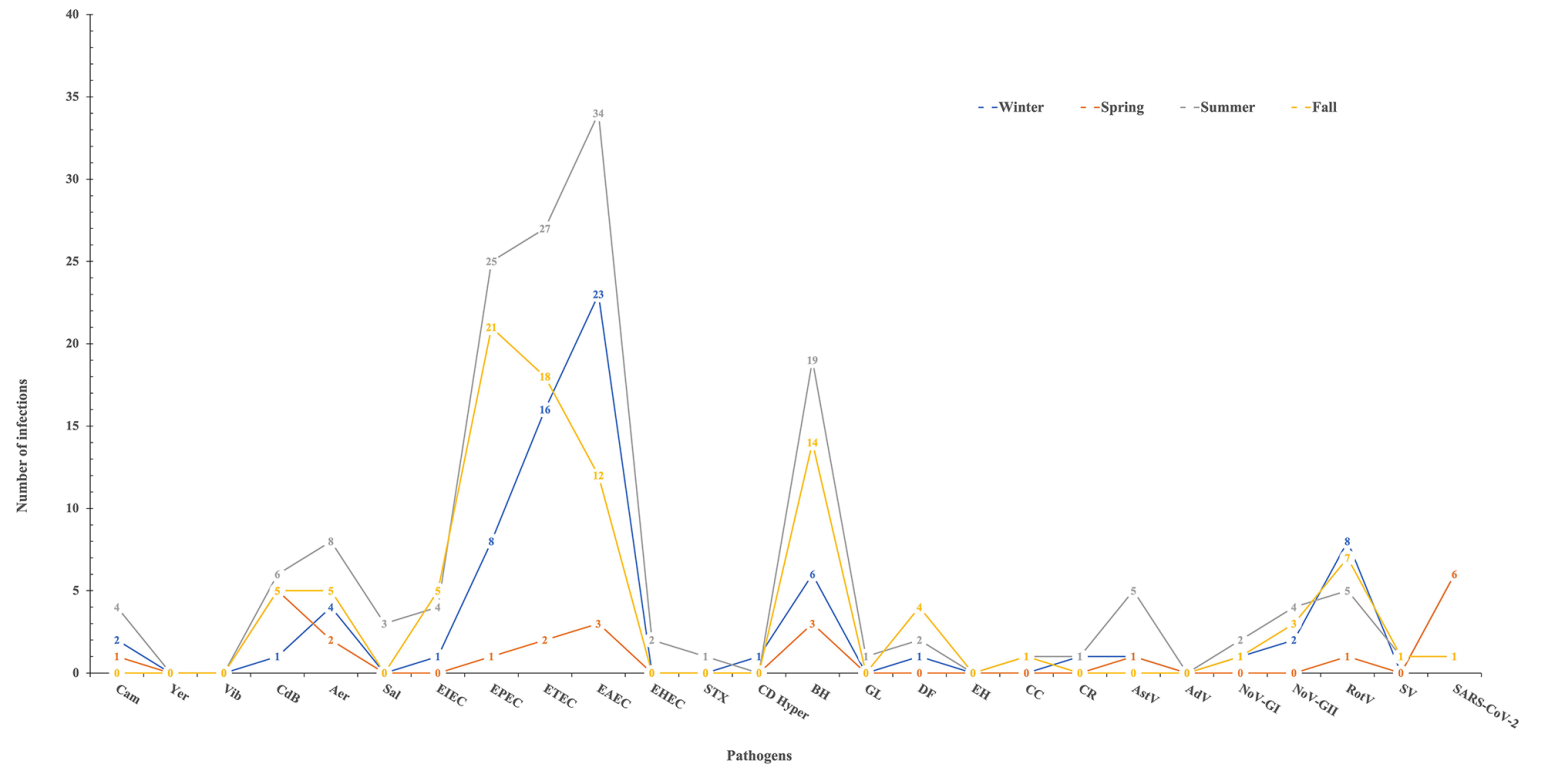
Seasonal distribution of enteropathogens detected from diarrheal stool

Enteric pathogens were prevalent during summer, fall and winter seasons (Fig. 5). Spring season had the lowest prevalence. No difference was observed based on the type of pathogens. Aer, EPEC, ETEC, EAEC, BH and RotV infections were reported throughout the year with the highest frequency of associated diarrhea episodes (79%, 98%, 83%, 79%, 81%, and 85% respectively) occurring during July and December. The prevalence of rotavirus showed a strong seasonal pattern. The seasonal curve of rotavirus had a peak in fall and winter. Astrovirus infections were highest in the summer. No seasonal trends were apparent for Cam, DF, NoV-GII, SARS-COV-2 (Fig. 5).

## Discussion

In our current study, implementation of multiplex PCR as a stool diagnostic test revealed a high positivity rate of 71% (192/271) for enteropathogens, 46% (88/192) as single infections and 54% (104/192) as mixed infections. Our study is consistent with previous study conducted in Lebanon which strengthens the validity of our findings, demonstrating the effectiveness of multiplex PCR for comprehensive pathogen detection [26]. A total of 29% of our samples (79/271) were negative; this could have been attributed to a non-infectious origin or immediate start of empiric treatment prior to sample collection and other idiopathic causes. A metagenomic approach for identification of pathogens associated with idiopathic diarrhea could be therefore useful to investigate negative samples [27–29].

Detection and characterization of diarrheagenic *E. coli* has not been well investigated in Lebanon. In our study, we have demonstrated a high rate of prevalence for EPEC, ETEC and EAEC with a high hospitalization rate caused by EPEC (43%) and ETEC (50%). Our results are comparable with the finding of another study published in Lebanon that has found that the prevalence of diarrheagenic *E. coli* in the population was as high as 70% [26]; it is worth mentioning that their sample population was collected mainly from the north of the country and distributed between rural and urban populations while the vast majority of our sample size came from in and around the capital and was more of urban in nature. The relatively high prevalence of diarrheagenic *E. coli*, observed in our study and in a recent publication of Osman et al. [26] express the importance of including EPEC, ETEC and EAEC in diagnosing the etiology of diarrheal disease. These bacteria can be acquired through maternal transmission, prior exposure, or geographic factors [30–32]. Some studies also suggested an environmental enteropathy in developing countries due to low income, low sanitation levels and unclean drinking water leading to the acquisition of colonizing diarrheagenic *E.coli* strains [33].

EPEC is only tested for children younger than 2 years. The pathogenic role of EPEC in children was described in previous studies since it is mostly isolated from diarrheal samples rather than controls leading to high dehydration rates of patients, and have a high mortality rate in hospitalized children [34–36]. However, in our study, EPEC and ETEC were also prominent in adult and elderly groups. This, in accordance with another study conducted in the United States, highlighting that EPEC burden in adults and elderly groups is high; these pathogens must be considered in adulthood diarrhea [37]. Further studies should be done with higher scale to investigate the role of these EPEC and ETEC consequently, the need of improving diagnostic methods. Integrating ETEC vaccination in young age groups should be also evaluated. EAEC was also observed in all age group which is in accordance with several previous studies that showed its presence in all age groups [36, 38]. The rate of hospitalization caused by EAEC was however lower than EPEC and ETEC as a single infection.

We reported an approximately even distribution of *C. difficile* between infants and adult group. *C. difficile* is widely detected in asymptomatic infants and its success in colonizing their intestinal tract is possible due to the undeveloped intestinal flora and consequent failure of resisting colonization [39–42]. Malignancies, hospitalization, and excessive antibiotic use can induce severe symptomatic infections in young children [43]. In this study, we supported that *C. difficile* is a colonizing bacterium in infants rather than a pathogenic agent since 43% of all *C. difficile* detected were found in infants and it was mostly detected with other intestinal pathogens in non-hospitalized patients.

One of the common practices at present is to test for toxigenic *C. difficile* in hospitalized patients. In the current study, 6.6% (16/244) of cases that were not screened for *Clostridium difficile* were positive by PCR. Currently, there are guidelines for the diagnosis of CDI state and an algorithm testing should be used, PCR should be therefore considered in testing since as stand-alone tests may be affected by their sensitivity/specificity.

It was notable that *Blastocystis hominis* was a very common agent observed in our study patients; it was detected in 15.5% (42/271). This pathogen is usually associated with diarrhea and abdominal pain [44]. Although, the prevalence of *B. hominis* seems high, it is still lower than what other studies conducted in Lebanon have observed [45–47]. This variation can be caused by the more urbanized nature of our sample population compared to others since most of our samples were collected from patients that reside in or around Beirut. Nevertheless, due to the economic situation as Lebanon transition from a high-middle-income country to a low- middle-income one, we expect this number to rise; the latter raises the importance of improving the detection of parasites such as *B.hominis* in the screening and diagnosis of diarrheal disease in Lebanon.Notably, our investigation contributes to the growing understanding of the role of *B. hominis* in diarrheal disease in Lebanon. These findings echo the study conducted by Osman et al. underscoring the consistency and relevance of our results in the broader context of gastrointestinal infections [48]. Improving diagnostic accuracy of this pathogen ultimately leads to better treatment outcomes and improved patient care. It reduces the risk of unnecessary treatments, minimizes the potential for complications, and ensures that patients receive the most appropriate interventions tailored to their specific condition.

Our study also highlights the vital contribution of several enteric viruses in gastroenteritis. The pathogen-disease association strategy is not a necessity in these enteric viruses due to their well- established pathogenicity and their rare incidence of colonization. In our study, the prevalence of rotavirus was found to be 25% (14/57) in children less than 5 years old and accounted for a hospitalization rate of 36% (5/14). Despite the rotavirus vaccine (Rotarix®) that is usually given to most of the pediatric population in Lebanon, the hospitalization rate is considered high. Although rotavirus had no significant effect on other age groups, the high prevalence of rotavirus in children, despite vaccination, can be explained by genetic drifts that can cause selective mutations in the virus. As a result, leading to concerns in the effective neutralization efficacy which highlight the importance of continuous screening and better diagnosis of rotavirus [49].

With the pandemic of SARS-CoV-2, it was shown that the virus can cause gastrointestinal symptoms and can be shed in stools after respiratory symptoms are resolved in nearly half of the patients infected [50–52]. Thus, we aimed to include SARS-CoV-2 in our GI panel; Our study has shown a 5.2% (14/271) positivity. Most of our SARS-CoV-2 positive samples were identified in the spring and winter seasons, during which there was a high rate of infection in Lebanon and an emergence of the delta variant. Further analysis should be done to identify the correlation between SARS-CoV-2 variants and GI symptoms.

Our findings align with previous studies demonstrating that coinfections are not associated with hospitalization [53–55]. However, it is important to note that our study may have limitations due to a relatively small sample size. To confirm these results and draw more conclusive evidence, further research with a larger scale and a higher number of hospitalized patients would be necessary. This would allow for a deeper understanding of the potential relationship between coinfections and hospitalization.

While our study aimed to provide an overview of gastrointestinal infections during the months of sample collection, we acknowledge the limitation associated with the unequal distribution of samples across seasons. Recognizing that gastrointestinal infections often exhibit seasonality, the modest sample size for each season limits the precision of our analysis. An unequal sample numbers per season was driven by the observed prevalence of these infections, which tend to be more common in warmer months. Furthermore, the variation in sample collection across different facilities may have influenced the observed percentages of pathogens. This study provides a foundation for understanding the presence of gastrointestinal pathogens during the sampled months, but a more comprehensive evaluation of seasonality would necessitate a larger and more evenly distributed sample across seasons and facilities. This limitation underscores the need for future research with expanded sample sizes to refine our understanding of the seasonal dynamics of gastrointestinal infections. Continued surveillance and accumulation of data over multiple years or across different geographical regions can help to gradually uncover and validate the seasonality patterns of pathogens with low prevalence.

The primary aim of this study was to assess the utility of implementing the multiplex PCR assay for the identification of enteric pathogens in Lebanon especially those traditionally challenging to identify using conventional diagnostic methods. Our results are consistent with the work of Osman et al., who utilized the BioFire FilmArray [26]. This alignment strengthens the validity of our findings, highlighting the effectiveness of multiplex PCR for comprehensive pathogen detection. As PCR techniques detect the presence of genetic material without distinguishing between live and dead organisms, it is crucial to acknowledge the potential limitation in attributing active disease solely based on positive PCR results. Therefore, a correlation between molecular findings with clinical data, patient symptoms, and other relevant diagnostic information should be considered prior to treatment.

There are a few limitations to this study. The absence of a control group that includes asymptomatic patients is a significant limitation. Comparing the colonization of the identified pathogens in symptomatic patients to asymptomatic individuals could provide valuable insights into the clinical relevance of the pathogen and its potential for causing disease.

Another limitation stems from the restricted sample size, a factor influenced by seasonal variations, financial constraints attributed to the economic crisis in Lebanon during that period, and the unforeseen impact of the COVID-19 pandemic. The small sample size may limit the generalizability of the findings and introduce selection bias. It is important to consider that the characteristics of the study population may not represent the broader population at risk.

Additionally, the lack of complete information on epidemiological variables for outpatients is a limitation. Without comprehensive data on these variables, it becomes challenging to evaluate clinically significant associations, particularly in relation to the severity of symptoms. It is essential to have a complete understanding of these factors in order to better characterize the impact of the pathogen on patients’ outcome.

## Conclusions

The adoption of multiplex real-time PCR assays in the diagnosis of gastrointestinal infections has identified gaps and improved the rates of detection for multiple pathogens. Our findings highlight the importance of conducting comprehensive surveillance to monitor enteric infections. The implementation of a syndromic testing panel can therefore provide healthcare professionals with timely and accurate information for more effective treatment and public health interventions.

### Electronic supplementary material

Below is the link to the electronic supplementary material.

Supplementary Material 1

Supplementary Material 2

Supplementary Material 3

## Data Availability

All data generated or analyzed during this study are included in this published article and its supplementary information files.

## Abbreviations

Aer: *Aeromonas spp*
Cam: *Campylobacter* spp
CdB: Clostridium diffcile toxin B
CD Hyper: Hypervirulent *Clostridium difficile*
Sal: *Salmonella* spp
Sh: *Shigella* spp
Vib: *Vibrio cholerae*
Yer: *Yersinia entercolitica*
EAEC: Enteroaggregative *Escherichia coli*
EPEC: Enteropathogenic *Escherichia coli*
EIEC: Enteroinvasive *Escherichia coli*
EHEC: Enterohemorrhagic *Escherichia coli*
ETEC: Enterotoxigenic *Escherichia coli*
STEC: Shiga toxin-producing *E. coli*
AdV: Adenovirus 40/41
NoV-GI: Norovirus GI
NoV-GII: Norovirus GII
RotV: Rotavirus A
AstV: Astrovirus
SV: Sapovirus
CR: Cryptosporidium spp
EH: *Entamoeba histolytica*
GL: *Giardia lamblia*
BH: *Blastocystis hominis*
DF: *Dientamoeba fragilis*
CC: *Cyclospora sayetanensis*
