# Supplementary material for: Enhancing enteric pathogen detection: implementation and impact of multiplex PCR for improved diagnosis and surveillance
Source: BMC Infect Dis. 2024 Feb 7;24:171. doi: 10.1186/s12879-024-09047-z (PMC10848388; doi:10.1186/s12879-024-09047-z)
Supplement: Supplementary file 1 — Supplementary Material 1 [file 12879_2024_9047_MOESM1_ESM.docx]

|  | **Pathogen** | **Abbreviation** | **Fluorophore labeled probe** |
| --- | --- | --- | --- |
| **Bacteria (n=13)** | *Aeromonas spp* | Aer | Quasar 670 |
|  | *Campylobacter spp* | Cam | FAM |
|  | *Clostridium diffcile toxin B* | CdB | Cal Red 610 |
|  | Hypervirulent *Clostridium difficile* | CD Hyper | Quasar 670 |
|  | *Salmonella spp* | Sal | Quasar 670 |
|  | *Shigella spp* | Sh | FAM |
|  | *Vibrio cholerae* | Vib | Cal Red 610 |
|  | *Yersinia entercolitica* | Yer | HEX |
|  | Enteroaggregative *Escherichia coli* | EAEC | Cal Red 610 |
|  | Enteropathogenic *Escherichia coli* | EPEC | FAM |
|  | Enteroinvasive *Escherichia coli* | EIEC | FAM |
|  | Enterohemorrhagic *Escherichia coli* | EHEC | Cal Red 610 |
|  | Enterotoxigenic *Escherichia coli* | ETEC | HEX |
|  | Shiga toxin-producing E. coli | STEC | FAM |
|  |  |  |  |
| **Viruses (n=6)** | Adenovirus 40/41 | AdV | HEX |
|  | Norovirus GI | NoV-GI | Cal Red 610 |
|  | Norovirus GII | NoV-GII | FAM |
|  | Rotavirus A | RotV | Quasar 670 |
|  | Astrovirus | AstV | FAM |
|  | Sapovirus | SV | Cal Red 610 |
|  | Coronavirus (ORF1ab, E and N genes) | SARS-CoV-2 | FAM, ROX, CY5 |
|  |  |  |  |
| **Parasite (n=6)** | *Cryptosporidium spp* | CR | Quasar 670 |
|  | *Entamoeba histolytica* | EH | Cal Red 610 |
|  | *Giardia lamblia* | GL | FAM |
|  | *Blastocystic hominis* | BH | FAM |
|  | *Dientamoeba fragilis* | DF | Cal Red 610 |
|  | *Cyclospora sayetanensis* | CC | Quasar 670 |
|  |  |  |  |
| Internal control |  | IC | HEX |

**Table S1: Summary of the Allplex™ full gastrointestinal assay panels**
