# Supplementary figures and images for: Enhancing enteric pathogen detection: implementation and impact of multiplex PCR for improved diagnosis and surveillance
Source: BMC Infect Dis. 2024 Feb 7;24:171. doi: 10.1186/s12879-024-09047-z (PMC10848388; doi:10.1186/s12879-024-09047-z)

**
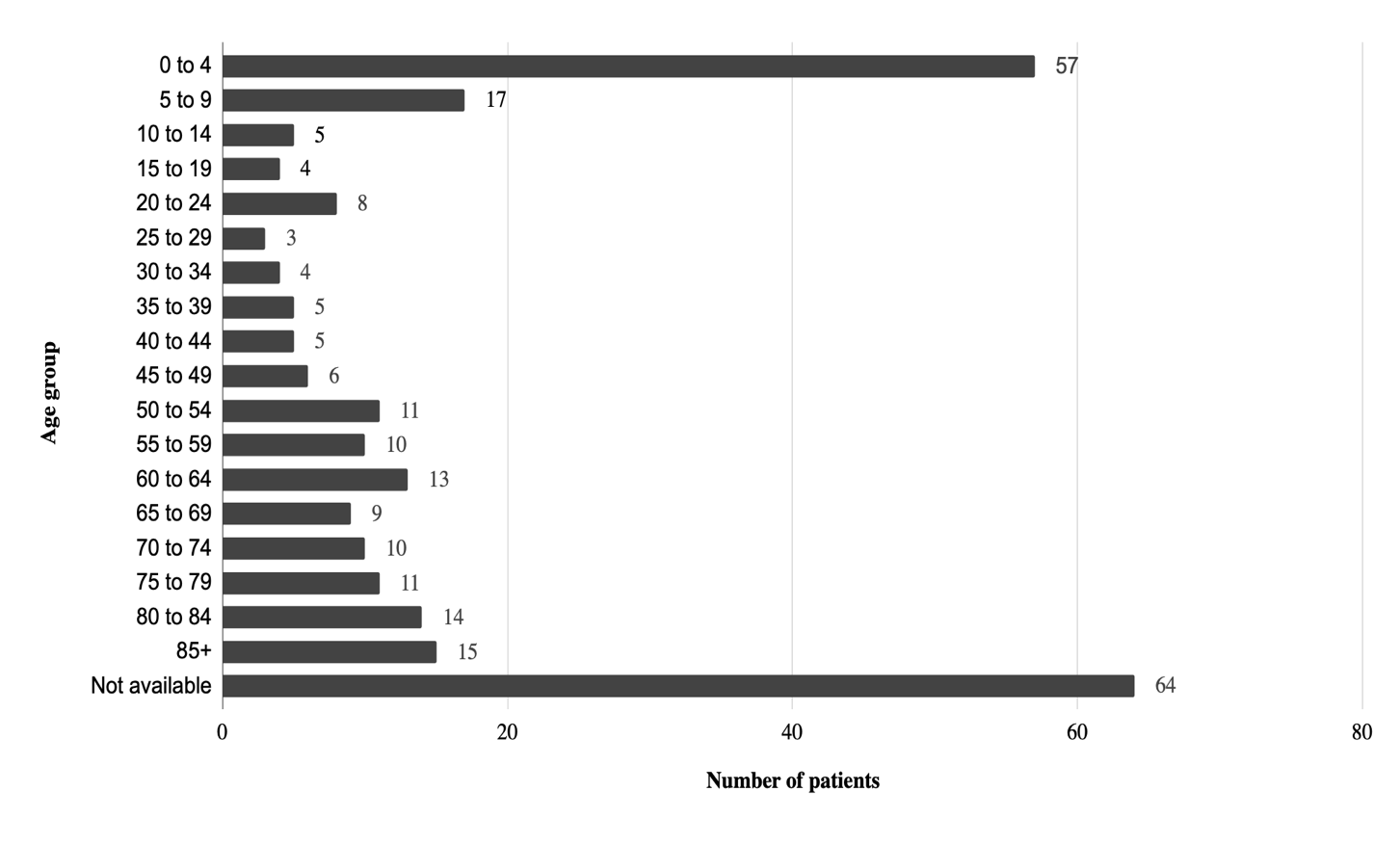
**

# Figure S1: Histogram showing the distribution of participants among different age groups.

Supplement: Supplementary file 2 — Supplementary Material 2 [file 12879_2024_9047_MOESM2_ESM.docx]
