# Supplementary material for: Enhancing enteric pathogen detection: implementation and impact of multiplex PCR for improved diagnosis and surveillance
Source: BMC Infect Dis. 2024 Feb 7;24:171. doi: 10.1186/s12879-024-09047-z (PMC10848388; doi:10.1186/s12879-024-09047-z)
Supplement: Supplementary file 3 — Supplementary Material 3 [file 12879_2024_9047_MOESM3_ESM.docx]

**Table S2: Total number of enteropathogens and coinfections detected in patients with acute diarrhea.**

In the first part of the table, we determined the total number of each pathogen and the concomitant presence of different pathogens. The number in bold (read vertically) indicates the total number of patients positive for each pathogen. Co-infections of pathogens were commonly observed (read horizontally).

In the second part of the table, coinfection, (read vertically), we describe the total number of each pathogen when detected as single pathogen or along with another co-infection.
